# Seasonal Phenology and Climate Associated Feeding Activity of Introduced *Marchalina hellenica* in Southeast Australia

**DOI:** 10.3390/insects14030305

**Published:** 2023-03-21

**Authors:** Duncan D. Jaroslow, John P. Cunningham, David I. Smith, Martin J. Steinbauer

**Affiliations:** 1 Department of Ecology, Environment and Evolution, La Trobe University, Melbourne, VIC 3086, Australia; 2 School of Applied Systems Biology, La Trobe University, Melbourne, VIC 3086, Australia; 3 Agriculture Victoria, AgriBio Centre for AgriBioscience, Melbourne, VIC 3086, Australia; 4 Agriculture Victoria, Biosecurity and Agricultural Services, Cranbourne, VIC 3977, Australia; 5 School of Ecosystem and Forest Sciences, University of Melbourne, Parkville, Burnley, VIC 3121, Australia; 6 ArborCarbon, Murdoch University, Murdoch, WA 6150, Australia

**Keywords:** seasonal phenology, phloem-feeders, insect density, honeydew, climate drivers

## Abstract

**Simple Summary:**

Giant pine scale, *Marchalina hellenica* Gennadius (Hemiptera, Marchalinidae), is a sap sucking insect native to the Eastern Mediterranean Basin. In 2014, giant pine scale was detected for the first time in Victoria, Australia, feeding on a new host, *Pinus radiata*. We studied the life cycle and feeding activity of giant pine scale in Victoria over 32 months, with the aim of drawing comparisons between exotic and native populations. Australian life stages of this pest emerged during similar months to Greek seasonal equivalents, although the timing of Australian life stages differed between years. Insect density and feeding activity on infested trees differed among locations and between generations. Strong evidence was found in support of density and feeding intensity being explained by climatic conditions. The value of *Pinus radiata* as a food source for this insect may fluctuate with climate conditions. Our findings aim to inform future management efforts for this scale insect, including surveillance strategies and optimal seasons for release of biocontrol agents. Our findings also suggest that the impact of this pest in Australia may be exacerbated by climate change.

**Abstract:**

Invasive insects pose an increasing risk to global agriculture, environmental stability, and public health. Giant pine scale (GPS), *Marchalina hellenica* Gennadius (Hemiptera: Marchalinidae), is a phloem feeding scale insect endemic to the Eastern Mediterranean Basin, where it primarily feeds on *Pinus halepensis* and other Pinaceae. In 2014, GPS was detected in the southeast of Melbourne, Victoria, Australia, infesting the novel host *Pinus radiata*. An eradication program was unsuccessful, and with this insect now established within the state, containment and management efforts are underway to stop its spread; however, there remains a need to understand the insect’s phenology and behaviour in Australia to better inform control efforts. We documented the annual life cycle and seasonal fluctuations in activity of GPS in Australia over a 32 month period at two contrasting field sites. Onset and duration of life stages were comparable to seasons in Mediterranean conspecifics, although the results imply the timing of GPS life stage progression is broadening or accelerating. GPS density was higher in Australia compared to Mediterranean reports, possibly due to the absence of key natural predators, such as the silver fly, *Neoleucopis kartliana* Tanasijtshuk (Diptera, Chamaemyiidae). Insect density and honeydew production in the Australian GPS population studied varied among locations and between generations. Although insect activity was well explained by climate, conditions recorded inside infested bark fissures often provided the weakest explanation of GPS activity. Our findings suggest that GPS activity is strongly influenced by climate, and this may in part be related to changes in host quality. An improved understanding of how our changing climate is influencing the phenology of phloem feeding insects such as GPS will help with predictions as to where these insects are likely to flourish and assist with management programs for pest species.

## 1. Introduction

Invasive insects pose a serious threat to forest health, ecosystem stability, and even public health in areas where they establish [1,2,3,4]. Field observations of an exotic insect’s life cycle can be important to understanding their population dynamics in the new environment and when control efforts could be maximised [5,6]. This would be particularly important for understanding species establishing in both the northern and southern hemispheres, due to the inverse of calendar season. Phenological studies might include documenting insect activity patterns and emergence of developmental stages (e.g., [7,8]) and, in the case of herbivorous insects, matching these to fluctuations in host quality [9,10]. For phloem feeding insects, mapping the timing of feeding life stages and feeding activity can be used to predict when impacts are greatest [11,12,13]. In Australia, insect forest pests are being detected at an accelerating rate [14,15], many of which are phloem-feeding insects.

In 2014, the giant pine scale, *Marchalina hellenica* Gennadius, (Hemiptera, Marchalinidae) was first detected in Victoria, Australia, feeding on the novel host *Pinus radiata*, or California Monterey pine [16]. *Marchalina hellenica* is endemic to the eastern Mediterranean basin [17,18,19]; its establishment on *P. radiata* (which originates from North America) poses a significant threat to Australian softwood plantations. *Pinus radiata* represents the vast majority of Australia’s more than 1 million hectares of softwood plantation [20]. Despite the widespread use of *P. radiata* as a plantation species and an ornamental, in public gardens and farm windrows, across Australia, the documented distribution of *M. hellenica* is currently restricted to southeast Melbourne [16].

Little is known about how the life cycle of *M. hellenica* in its new Australian habitat compares to its endemic range. The insect’s three immature nymphal instars acquire nutrients for development from the sap of pine trees [21]. The nymphal instars feed on phloem by inserting syringe-like stylets into twigs, branches, trunks, and exposed roots [22,23,24]. As with many phloem-feeding insects, excess carbohydrates and undesirable compounds such as insecticides [25] that are ingested must be excreted as honeydew [26,27]. *Marchalina hellenica* does not feed on host trees all year round, and only the nymphal instar stages possess a functioning stylet and produce honeydew [28]. *Marchalina hellenica* likely originates from Mount Carmel in Northern Israel, the origin of its primary host, *P. halepensis* [29]. Honeydew produced by *M. hellenica* is collected by honeybees across the Eastern Mediterranean and accounts for 60% (~15,000 tonnes) of annual honey produced in Greece [30,31]. While this is important for apiculture in Greece, it also highlights the phloem feeding capability of this insect. Its impact as an introduced pest to Australian forestry could be severe, particularly if feeding stages are prolonged and/or populations flourish in the absence of important native predators, such as the predatory fly, *Neoleucopis kartliana* Tanasijtshuk (Diptera: Chamaemyiidae) [32]. In addition to exuding honeydew, *M. hellenica* secretes cotton-like wax filaments [21] termed flocculent, which may provide a hydrophobic layer and microclimate to protect scales from desiccation and flooding of feeding sites [33,34,35].

Insect density and availability of honeydew are key indicators of the resources expropriated by *M. hellenica*. Explaining variation in these indicators is important for understanding the insect’s activity patterns. As an ectothermic phloem feeder, *M. hellenica* activity is likely related to climate via effects on host nutritional quality [36,37]. As an insect sheltering under bark and flocculent, climate conditions directly experienced by *M. hellenica* may therefore provide inferior explanations for variation in insect activity than the climate experienced by host plants [9,38]. Understanding how *M. hellenica* responds to the climate may help predict the potential spread of this pest in Australia or the impact as the climate in the Mediterranean [39,40] and Pacific region [41,42] trend towards more unpredictable conditions through climate change. 

In this study, we used field-sampling to document the seasonal phenology of *M. hellenica* in southeast Melbourne, Victoria, across two and a half insect generations. As these represent the only known *M. hellenica* populations in the southern hemisphere or outside Europe, we explored whether major life cycle milestones (e.g., nymph emergence or moults) in Melbourne aligned with equivalent seasonal periods to East-Mediterranean conspecifics (i.e., 6 calendar-month difference). We also investigated whether the timing of instar stages and honeydew production (as the proportion of honeydew producing insects, HPI) followed the same seasonal pattern as Mediterranean conspecifics. As a univoltine (one generation per year) and semelparous (mortality following oviposition) insect, density of *M. hellenica* was predicted to reach the generational minimum during the adult stage and maximum at nymph emergence. Finally, we explored the prediction that variation in *M. hellenica* activity would be best explained by the environmental conditions experienced by the host tree, rather than the conditions directly experienced by *M. hellenica*.

## 2. Materials and Methods

### 2.1. Study Sites

Infested *M. hellenica* branches were collected every second week (fortnightly) from January 2019 to October 2021, or weekly during moulting periods, from two field sites in south-east Melbourne, Australia: Cardinia Reservoir Park (CRP) in Cardinia (managed by Parks Victoria, a government agency of the state of Victoria, Australia) (37°58′1.5″ S, 145°23′41.4″ E), and Dalton Reserve (DR) in Harkaway (managed by City of Casey Council) (38°0′8.3″ S, 145°20′35.2″ E). Insects were considered to be in a moulting period when more than one life stage was concurrently detected.

The two field sites were selected due to (i) the reliable accessibility, (ii) trees not being removed during the three-year survey period, and (iii) differences in habitat structure, specifically tree density and size. *Pinus radiata* trees at CRP were planted in 1973 to help stabilise soil around the reservoir’s banks [43]. The stands of *M. hellenica* infested *P. radiata* were planted in plantation style rows and are of a fairly uniform age. The understory is smothered in dead *P. radiata* needles, with little to no grass or other ground-covering vegetation and an open mid-story consisting of native *Pittosporum undulatum*. Forest surrounding these *P. radiata* stands were dominated by *Eucalyptus* and *Acacia* species. By contrast, DR is a small publicly accessible park located in Harkaway. There are only a handful of ornamental *P. radiata* at DR, all of which were found to be infested. The DR pine trees were much larger than those sampled at CRP. The ground of this reserve is covered by introduced grasses. Other large ornamental trees are present, such as *Eucalyptus leucoxylon* and *Acacia mearnsii*; the canopies of *P. radiata* and other trees at DR do not form a closed canopy. 

### 2.2. Environmental Conditions

Local temperature and relative humidity (RH %) at CRP were recorded using data loggers (accurate to 1.0 °C and resolved to 0.125 °C; DS1923, iButton^®^, Whitewater, WI, USA). At each site, a data logger was either affixed to the surface of an infested tree trunk (exposed) or wedged into a *P. radiata* bark fissure and under naturally present flocculent, where sufficient flocculent was present to shelter the data logger (fissure) as close to 1.5 m above the south-facing base of the tree as feasible. Data loggers recorded temperature and relative humidity every 30 min, with the exception of a few recordings missed during monthly data retrieval and logger resets. Vapour pressure deficit (VPD), recorded in kPa, was calculated from temperature and relative humidity via an established adaptation of the Magnus equation for vapour saturation point [44,45,46]. Exposed records were recorded over 707 days (1 November 2019–7 October 2021) and bark fissure records over 469 days (26 June 2020–7 October 2021).

Temperature (°C) and solar exposure (MJ m^−2^) records were sourced from the Bureau of Meteorology and recorded at Ferny Creek weather station [47]. The Bureau of Meteorology sourced records are referred to here as ‘ambient’ climate variables. Daily rainfall (mm) was sourced from Melbourne Water [48] and recorded in Emerald, which was closer to field sites than Ferny Creek weather station and at a similar elevation. Rainfall, solar exposure, and ambient temperature records span the entirety of the honeydew and density data; 934 days (19 March 2019–7 October 2021). This also allowed temperature from iButtons to be compared with ambient temperatures captured by weather stations. Daily climate maximums were converted to fortnightly means for analyses. Rainfall was recorded as cumulative rainfall per fortnight.

The three climate record types covered different temporal ranges. To better compare climate record type, ambient and exposed climate recordings were truncated to match the time period recorded by fissure records (26 June 2020–7 October 2021; 469 days).

### 2.3. Seasonal Phenology

**Figure 1:**
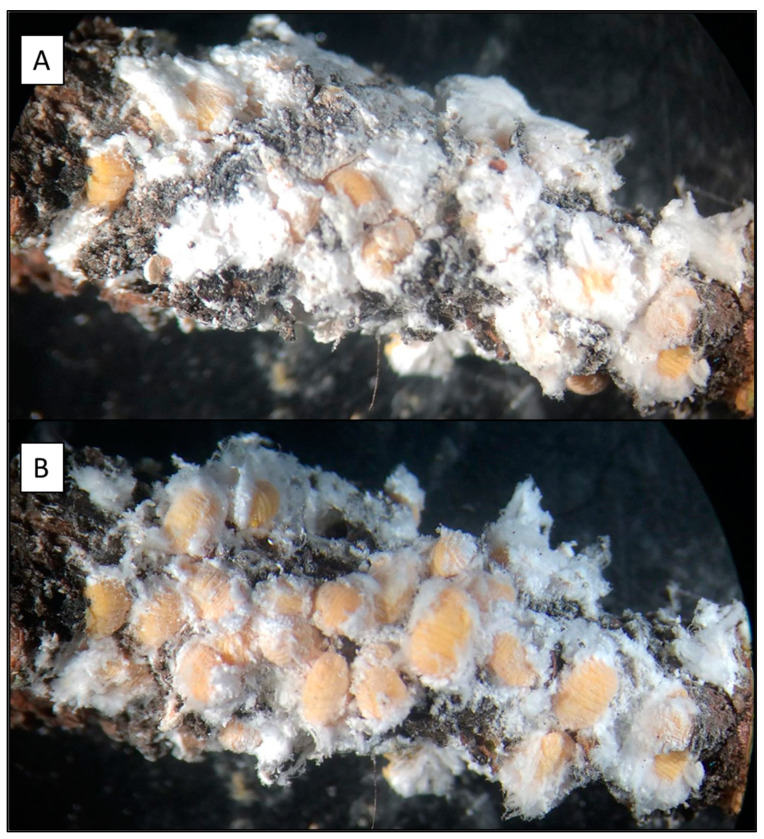
*Pinus radiata* twig, collected from CRP in March 2019, measuring 1.6 cm × 4.0 cm, and infested with 2nd instar stage *Marchalina hellenica*. Images show the same infested twig after excising (**A**) and after flocculent was removed (**B**). In view are 34 individual 2nd instar nymphs at a density of 0.60 GPS cm^−2^.

Seasonal phenology was documented at a minimum interval of fortnightly from January 2019 to October 2021. *Pinus radiata* infested with *M. hellenica*, since December 2018, at each field site were recorded. *Pinus radiata* trees were randomly selected for sample collection through a series of dice rolls. *P. radiata* were excluded from this random selection if their stem diameter was less than 50 cm or if branches were not accessible from ground-level. Sampled *P. radiata* at CRP were approximately 75-metres apart and 140-metres apart at DR, meaning the foliage of these trees at each site did not touch. Excised branches were collected from two trees infested with *M. hellenica* at each site across the study period. Samples were limited to two samples of 30 cm of infested branches per tree per week, with a diameter from 0.5 cm to 2 cm. Infested branches were those that had fresh flocculent or wandering adults (for September to October). Lengths of 9 cm to 11 cm subsections of infested pine twigs and *M. hellenica* were inspected in the laboratory under a dissection microscope (Wild M3Z, Wild Heerbrugg, Heerbrugg, Canton of St. Gallen, Switzerland) (Figure 1) and illuminated using a halogen gooseneck lamp (KL 2500 LED, Leica, Wetzlar, Hesse, Germany). Surface area of sampled twigs, calculated from twig length and diameter, was used to determine the density of *M. hellenica*, expressed as *M. hellenica* per square centimetre (GPS [cm^−2^]) [49].

The developmental stage of *M. hellenica* present on host material was determined based on a combination of antennal segment count, presence of body setae or dermal spines, and sclerotized body part size (key to *M. hellenica* life stages: [28]). Life stages were identified as honeydew producing insects (HPI) via visual confirmation of the presence of honeydew after lightly squeezing their abdomen [50]. The number of HPI was converted to a percentage of the sampled population of *M. hellenica* (HPI %). To account for combined variation in GPS [cm^−2^] and HPI [%], GPS [cm^−2^] was multiplied by HPI [%] to obtain the density of honeydew producing insects, expressed as HPI [cm^−2^]. This was used as an estimation of the density of *M. hellenica* producing honeydew, accounting for the decline in GPS [cm^−2^] as each generation progresses [51] and seasonal fluctuations in HPI [%] [50]. Honeydew was collected from HPI from each phenological sample. When less than 10 HPI were available, all HPI were recorded. During the honeydew squeeze test of these HPI subsets, any honeydew was collected using microcapillary tubes of known volume, enabling honeydew volume (HV μL) to be calculated. GPS [cm^−2^], HPI [%], HPI [cm^−2^], and HV [μL] values were converted to fortnightly means for each field site. For GPS [cm^−2^], 61 fortnightly samples for CRP and 50 for DR were retained for analysis. CRP had 49 and DR had 44 HPI [%] fortnights sampled, allowing HPI [cm^−2^] to be calculated across 49 and 44 fortnights for CRP and DR, respectively. CRP had 26 and DR had 27 fortnights sampled for mean HV [μL].

### 2.4. Statistical Analysis

Statistical analyses were carried out in SPSS (version 28.0.1.0, IBM Corp., Armonk, NY, USA; access provided by La Trobe University: Department of Ecology, Environment and Evolution; α = 0.05) [52]. Life cycle milestones for recorded generations were reported descriptively, including the first detection of each life stage within generations. The proportions of instar, adult, and egg stages represented in sampled populations were plotted over time, with HPI % overlayed to facilitate visual interpretation of *M. hellenica* phenology in relation to honeydew availability [50,53]. Maximum and minimum values for GPS [cm^−2^], HPI [%], HPI [cm^−2^], and HV [μL] were reported across *M. hellenica* generation and collection sites. Pearson’s correlation was used to determine which instar stage had the strongest correlation with HPI [%]. Fortnightly means of insect density across both CRP and DR were grouped by calendar month, which were then compared for differences in GPS [cm^−2^] via Kruskal–Wallis Test of Independent Samples (KW). Differences between specific months included in KW were compared for temporal differences via Dunnett’s all-pairs comparison test (Dunnett T3). Dunnett T3 is a non-parametric post-hoc test, and thus, does not assume equal distribution or variance among groups [54]. Dunnett T3 was unable to resolve differences in insect density between any month-based groups. To indicate which months were most dissimilar, the Tukey’s Honestly Significant Differences (Tukey’s HSD) post-hoc test was used to explore differences in density between months [55], although Tukey’s HSD outcome must be interpreted with caution (Tukey’s HSD assumes data are parametric). 

Differences in fortnightly means of *M. hellenica* activity between site and generation groups were tested via ANOVA. Where ANOVA was used, Tukey’s HSD was used for post-hoc comparison of differences among groups. Where data were determined to be non-parametric via Levene’s test of equal variances among groups, differences between site and generation groups were tested via KW. Group comparison via pairwise independent samples *t*-test where KW was used did not assume equal variances between groups. This process was done for GPS [cm^−2^], HPI [cm^−2^], and HV [μL]. HV [μL] was also compared across instar stages; however, here, individual insect honeydew volume is used, rather than fortnightly means. The natural log (ln(*x*)) of HV [μL] was used instead of the untransformed HV [μL] values, as this enabled better clarification of differences between instar stages via KW and between generations and field sites via MWU.

Fortnightly climate means were used in regression analyses to predict GPS [cm^−2^], HPI [%], HPI [cm^−2^], and HV [μL] recorded fortnightly samples. HPI [cm^−2^] and HV [μL] were ln(*x*) transformed prior to regression analysis, as this provided stronger climate models that were a better fit for the tested data. Climate variables included temperature (°C), RH (%), VPD (kPa), as recorded by exposed and bark fissure data loggers, and ambient temperature, cumulative rainfall (mm), and solar exposure (MJ m^−2^). Statistics associated with these models may be found in the Appendix A Table A9, Table A10, Table A11 and Table A12. Indicators of data fit, correlation strength, and statistical clarity for regression models were used to draw comparisons between models: for instance, if the same climate variable would explain variation in insect activity when recorded from different data sources (i.e., bark fissure, exposed trunk, weather station). Models for predicting HPI [cm^−2^] or HV [μL] were plotted for each of the climate variables sources from the data recorder that produced a statistically significant regression model with the strongest correlation strength. The relationship between HPI [cm^−2^] or HV [μL] and solar exposure (MJ m^−2^) was also plotted, although solar exposure (MJ m^−2^) was only sourced from the Bureau of Meteorology. Approximate Nonlinear Durbin–Watson test was used to check for autocorrelation among HPI [cm^−2^] or HV [μL] and climate variables [56,57].

**Figure A1:**
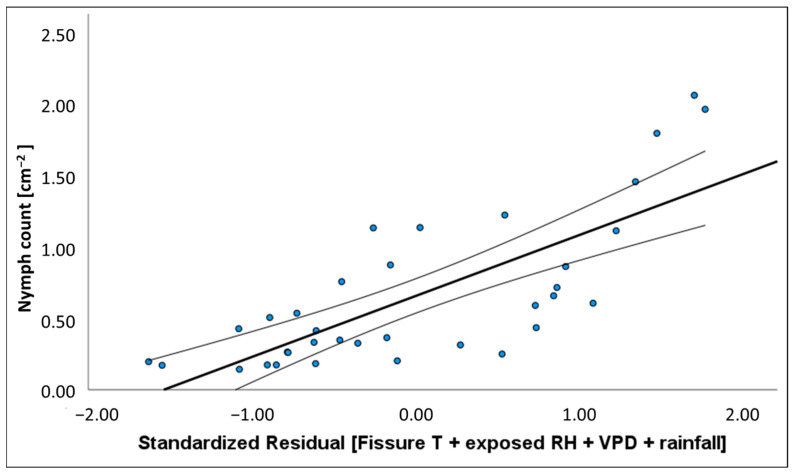
Scatterplot of *Marchalina hellenica* nymph density (cm^−2^) plotted over standardised residual of best climate predictor variables. Regression model is plotted as continuous black line, and curved black lines indicate 95% confidence intervals of the mean. Climate variables were selected if they provided the strongest model identified for a given climate variable. Summary: *p* < 0.001, F_1,33_ = 44.439, *r*^2^ = 0.574; model equation: Y = 0.427*x* + 0.656.

**Figure A2:**
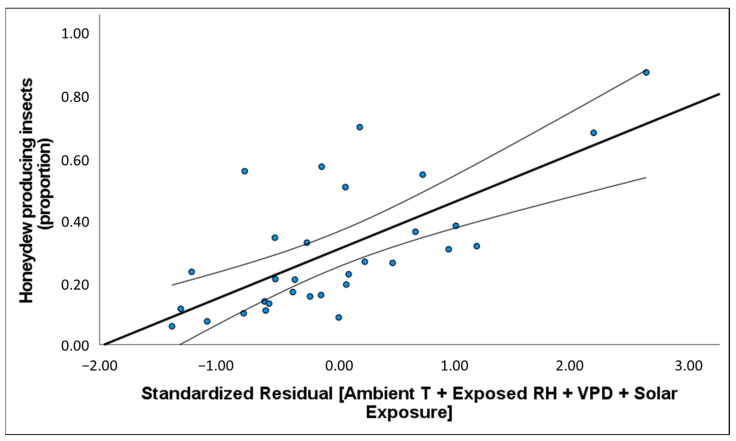
Scatterplot of the percentage of honeydew producing *Marchalina hellenica* plotted over standardised residual of best climate predictor variables with linear regression model plotted as curved black line. Curved black lines indicate 95% C. I. of the mean. Climate variables were selected if they provided the strongest model identified for a given climate variable. Summary: *p* < 0.001, F_1,29_ = 26.031, *r*^2^ = 0.473; model equation: Y = 0.153*x* + 0.3.

**Figure A3:**
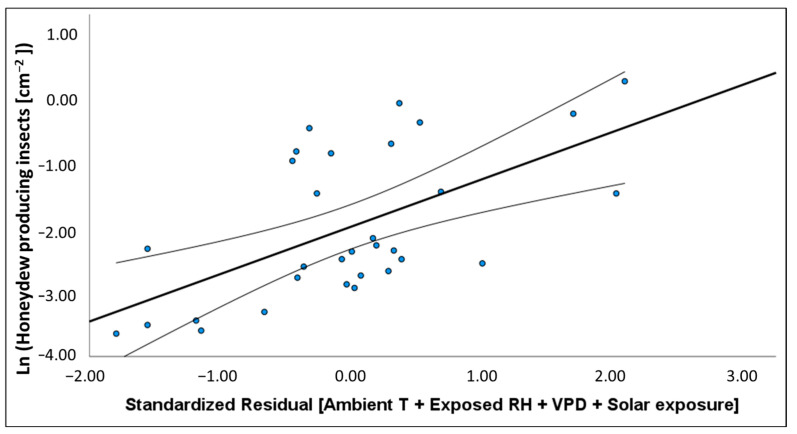
Scatterplot of log transformed density of honeydew producing *Marchalina hellenica* (cm^−2^) plotted over standardised residual of best climate predictor variables with exponential regression model plotted as curved black line. Curved black lines indicate 95% confidence interval of the mean. Climate variables were plotted if they provided the strongest model identified for equivalent climate variable. Summary: *p* < 0.001, F_1,29_ = 15.814, *r*^2^ = 0.353; model equation: Y = 0.727 × log(*x*) − 2.008.

**Figure A4:**
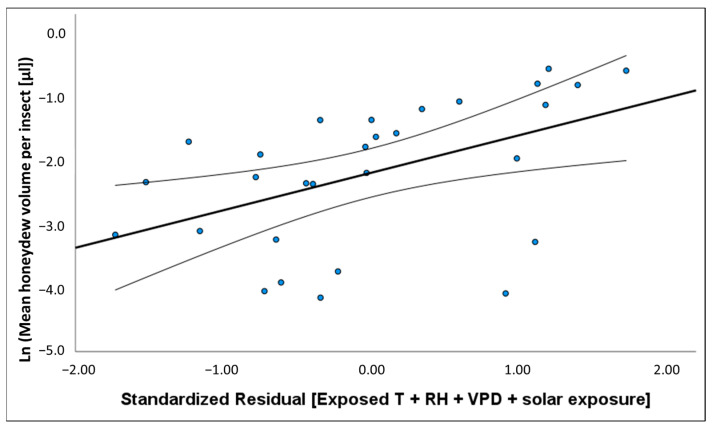
Scatterplot of log transformed mean honeydew volume excreted by *Marchalina hellenica* (μL) plotted over standardised residual of best climate predictor variables with exponential regression model plotted as curved black line. Curved black lines indicate 95% confidence interval of the mean. Climate variables were plotted if they provided the strongest model identified for equivalent climate variable. Summary: *p* = 0.008, F_1,27_ = 8.196, *r*^2^ = 0.233; model equation: Y = 0.583 × log(*x*) − 2.215.

Climate variables explaining a particular *M. hellenica* activity were included in multiple regression analysis with the goal of obtaining residual values for all climate variables included. Since climate variables were measured with differing units of measurement, standardised residual values were extracted. These standardised residual values were then included in a linear regression analysis to confirm that a given *M. hellenica* was responsive to the combined effect of the temperature (°C), RH (%), VPD (kPa), and solar exposure (MJ m^−2^) records that most strongly correlated with *M. hellenica* activity. These standardised residual plots, with 95% confidence intervals of the mean, are visualized in Figure A1, Figure A2, Figure A3 and Figure A4.

## 3. Results

### 3.1. Seasonal Phenology

**Figure 2:**
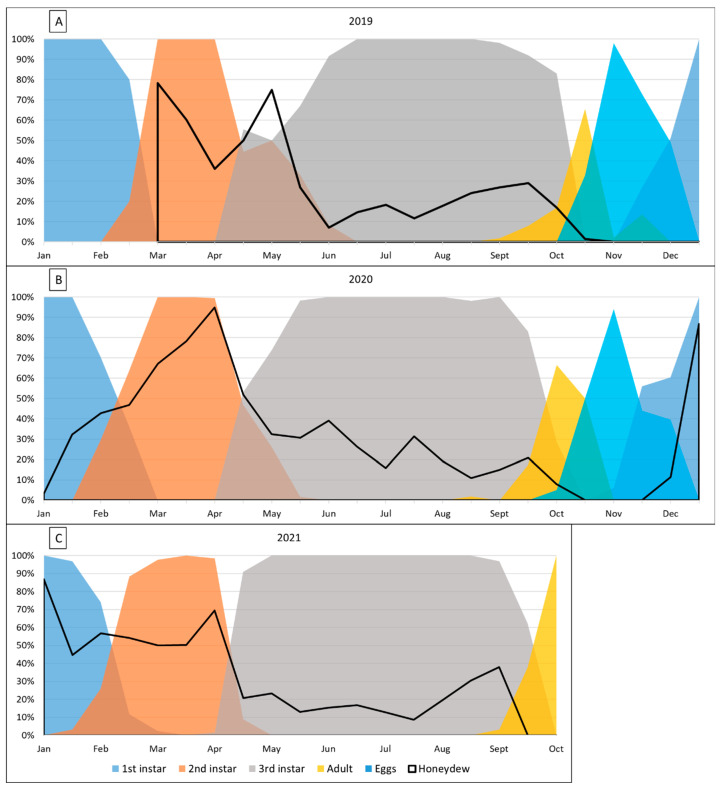
Phenology of *Marchalina hellenica* in Southeast Melbourne, showing the abundance of each life stages as a proportion of population in (**A**) 2019, (**B**) 2020, and (**C**) 2021, and the proportion of insects producing honeydew (black line).

The onset and duration of seasonal feeding behaviour was visualized from January 2019 (Figure 2A), 2020 (Figure 2B), and up to October 2021 (Figure 2C). *Marchalina hellenica* 1st instar nymphs largely emerged from their eggs and ovisacs in late November (Figure 2A). First detection of instar stage emergences occurred progressively earlier with each year (Table A1), although not for the adult stage. In 2019, the first instars began moulting in February, but the first instars began moulting in late January and early February in 2020 and 2021. The persistence of the second instar appeared to shorten each year; it was present for 17 weeks in 2019, 16 weeks in 2020, and 12 weeks in 2021. The third instars were always initially detected in April. However, the time from the first time that the third instar was detected until 100% of insects sampled were third instars, was less in 2021 than earlier generations. The third instar stage was the longest persisting stage, lasting up to 7 months from April until October. Final ecdysis usually occurred around mid-September when the third instars moulted into adults. By mid-October 2019 and 2020, the third instars were absent.

Population progression also appeared to accelerate each year for adults. For populations sampled in mid-September, 8% of *M. hellenica* were adults in 2019, 17.24% in 2020, and 37.79% in 2021. Two adults were detected in mid-late August 2020, although none were found the next fortnight or in August of 2019 and 2021. Despite most adults being present during October, they may persist into November. Adults oviposit throughout late September and October, with eggs developing for approximately 4–6 weeks before emerging into first instar nymphs around late-November. Adult *M. hellenica* are semelparous organisms, meaning that they die after a single reproduction event. As a result, by the time the first instars emerge, the adults have almost entirely perished.

### 3.2. Feeding Activity

Data collection of honeydew producers in samples did not commence until 21 March 2019; hence, a sudden spike in honeydew availability was visualized in Figure 2A. Insects emerging in November 2019 were not detected as producing honeydew until early January 2020 (Figure 2B), compared to nymphs emerging November 2020, which commenced honeydew production in early December 2020 (Figure 2B). HPI [%] was significantly correlated with the proportion of second instar nymphs in a population (Pearson correlation, *r* = 0.747, *t* = 7.368, *p* < 0.001). As a percentage of the sampled population, the second instar stage was found to be most the likely to produce honeydew compared to all other life stages observed (Table A2). The exception to this was a two-week period from late December 2020 to early January 2021 when 86.80% of the first instar nymph population were producing honeydew (Figure 2C). HPI [%] did not exceed this percentage again in 2021, with 69.35% being the next highest in 2021, recorded from second instar nymphs in early April. As insects reach their second ecdysis, honeydew production decreased sharply in all generations. Third instar nymphs were much less likely to produce honeydew than second instar nymphs, with third instar HPI [%] never exceeding 50% during winter months. In all generations, there was a small but noticeable rise in HPI [%] from late August to early September of 2019 and 2021.

**Figure 3:**
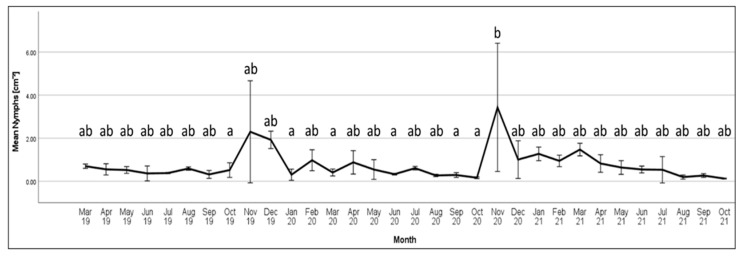
Seasonal mean density of *Marchalina hellenica* nymphs, combining samples from *Pinus radiata* at Dalton Reserve and Cardinia Reservoir Park across 32 months (±2 S.E). Significant differences are indicated by groups that do not share a letter (Tukey’s HSD; *p* < 0.05). N ≥ 2 for March–July 2019, August 2020, and October 2021, N ≥ 4 for all other groups.

Analysis of *M. hellenica* density across 32 months revealed differences across temporally separated populations (Kruskal–Wallis test of independent samples (KW): *p* = 0.023, F_31,85_ = 48.590). Variability in mean density was visualized in Figure 3. Density was highest during November 2020, when *M. hellenica* first instar nymphs were emerging from eggs. Tukey’s HSD provided evidence of density-related differences between specific temporal groups (Table A8), but since this test assumes a normal distribution and equal variances among groups, the outcome must be interpreted with caution. Most months could not be distinguished, with only November 2020 differing from a few months of 2020 and October 2019.

**Figure 4:**
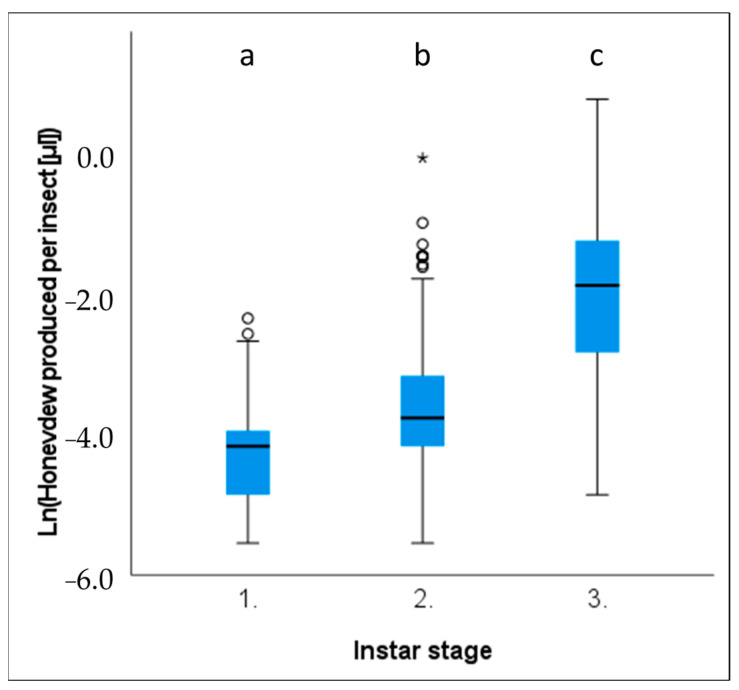
Box and whisker plot of honeydew volume (natural log transformed) produced per *Marchalina hellenica* nymph (ln (HV [μL])). Box and whisker boxes represent interquartile ranges, with the horizontal black line within the boxes indicating the group mean. Outliers are indicated by circles and asterisks. Significant differences between groups are indicated by different letters (*p* < 0.05).

Ln(*x*) transformed volumes of honeydew produced per insect (HV [μL]) were found to increase with each instar stage (Kruskal–Wallis: *p* < 0.001, F_2,454_ = 189.592*,* Figure 4). Pairwise comparisons of each instar stage demonstrated significant differences between all group pairings, with HV [μL] production exhibiting an exponential increase as each successive instar stage is reached (Figure 4; Table A3). First and third instar stages demonstrated the most substantial difference in honeydew produced per insect (Table A4). First instars produced a mean volume of 0.019 μL, and the third instars produced a mean of 0.240 μL, which is approximately twelve-times more honeydew.

**Figure 5:**
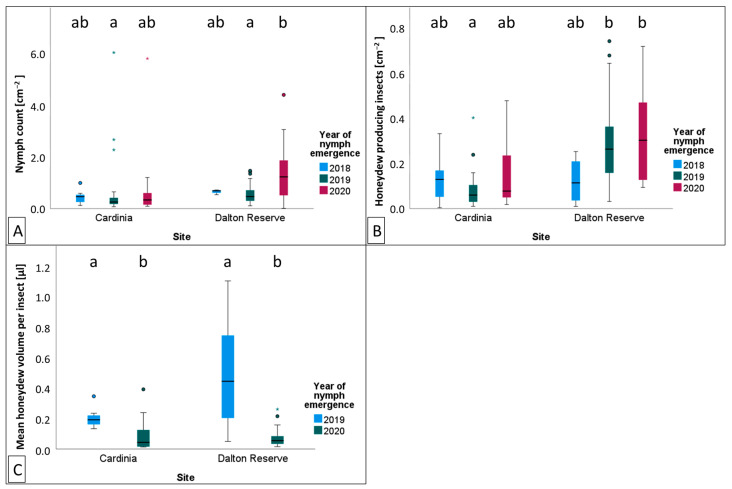
Box and whisker plots of *Marchalina hellenica* population activity at Cardinia or Dalton Reserve during the 2018, 2019 or 2020 emergent generations. *Marchalina hellenica* activity illustrated includes (**A**) nymph count per cm^2^, (**B**) honeydew producing insects per cm^2^, and (**C**) mean honeydew volume excreted per insect [μL]. Box and whisker boxes represent interquartile ranges, with the horizontal black line within the boxes indicating the group mean. Outliers are indicated by circles and asterisks. Significant differences between groups are indicated by different letters (*p* < 0.05).

Differences were identified between site and generation groups for GPS [cm^−2^] (ANOVA: *p* = 0.027, F_5,106_ = 2.647), HPI [cm^−2^] (KW: *p* < 0.001, F_5,93_ = 25.822), and HV [μL] (KW: *p* < 0.001, F_3,53_ = 24.348; Table A5, Table A6 and Table A7). GPS [cm^−2^] recorded at Dalton Reserve (DR) across 2020 was approximately 144% greater than the generation emerging at DR in 2019 (Tukey’s HSD: *p* = 0.039, N = 47; Figure 5A). HPI [cm^−2^] was approximately 242% greater at DR than Cardinia Reservoir Park (CRP) in 2019 (KW pairwise comparison: *p* < 0.001, *t* = 25.822, N = 93; Figure 5B). HV [μL] produced by *M. hellenica* was reduced for the 2020 generation compared to 2019 for both sites (Figure 5C). HV [μL] was approximately 55% lower in 2020 than 2019 at CRP (KW pairwise comparison: *p* = 0.024, *t* = 2.876, N = 26) and approximately 84% lower in 2020 than 2019 for DR (KW pairwise comparison: *p* < 0.001, *t* = 3.927, N = 27).

### 3.3. Insect Activity Response to Climate

Climate variables used to predict honeydew producer insects per cm^−2^ (HPI [cm^−2^]) generally provided stronger and more statistically significant models compared to either GPS [cm^−2^] or HPI [%] alone. This may be due to HPI [cm^−2^] being a derivative accounting for both metrics. For this reason, only climate models specifically predicting HPI [cm^−2^] and honeydew volume produced per insect (HV [μL]) are reported here. Only the strongest model for each climate variable was reported. Other model summaries of each *M. hellenica* activity variable and each climate record type are available in the Appendix A (Table A9, Table A10, Table A11 and Table A12). AND test did not reveal evidence of autocorrelation between climate variables and ln (HPI [cm^−2^]), or climate variables and ln (HV [μL]), for statistically significant regression models (Table A11 and Table A12).

**Figure 6:**
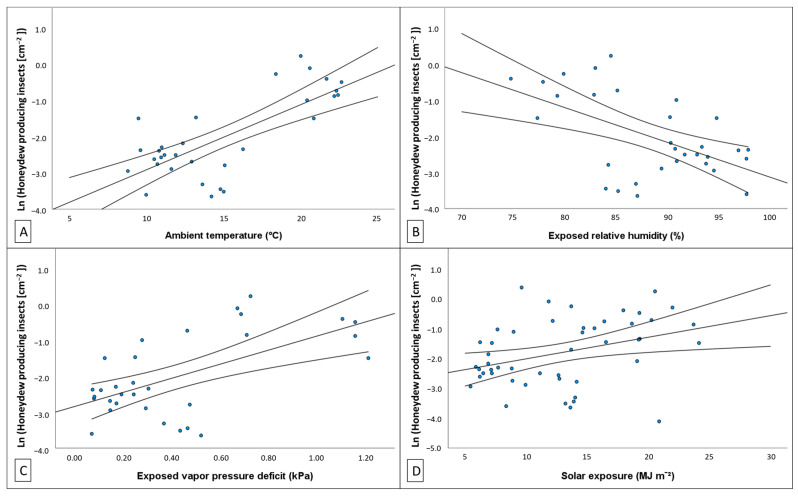
Relationship between natural log transformed fortnightly density of honeydew excreting nymphs (ln (HPI [cm^−2^])) to (**A**) exposed temperature, (**B**) exposed relative humidity, (**C**) exposed vapor pressure deficit, and (**D**) solar exposure. Regression model is plotted as continuous black line, and curved lines indicate confidence intervals (95%) of the mean. Strong evidence was found that multiple climate variables correlate with, and explain, observed variation in ln (HPI [cm^−2^]) (Table A11).

Statistically significant trends were found clarifying that ln (HPI [cm^−2^]) was at least weakly correlated with most climate variables (Table A11). As temperature increased, HPI [cm^−2^] increased exponentially. Ambient temperature provided the strongest model correlation of this relationship (linear regression: *p* < 0.001, F_1,29_ = 35.231, *r*^2^ = 0.549; Figure 6A) and indicated that increases in ambient temperature may explain approximately 53.3% of observed variation in ln (HPI [cm^−2^]). Statistical evidence found that ln (HPI [cm^−2^]) was negatively related to exposed RH, which explained 27.9% of the variation observed in HPI [cm^−2^] (linear regression: *p* = 0.0013, F_1,29_ = 12.858, *r*^2^ = 0.303; Figure 6B). Natural log transformed HPI [cm^−2^] was moderately correlated with exposed VPD (linear regression: *p* < 0.001, F_1,29_ = 16.317, *r*^2^ = 0.360; Figure 6C), explaining approximately 33.8% of variation observed in HPI [cm^−2^]. Higher solar exposure was found to be associated with exponential increases in HPI [cm^−2^] (linear regression: *p* = 0.015, F_1,29_ = 6.394, *r*^2^ = 0.122; Figure 6D). This was a weak correlation, with variation in solar exposure explaining approximately 10.3% of observed variation in HPI [cm^−2^].

**Figure 7:**
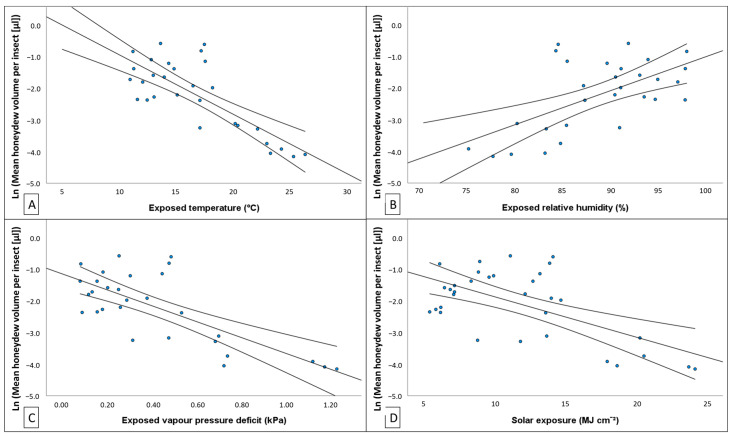
Relationship between natural log transformed fortnightly *Marchalina hellenica* honeydew excreted per insect (ln (HV [μL])) to (**A**) exposed temperature, (**B**) exposed relative humidity, (**C**) exposed vapor pressure deficit, and (**D**) solar exposure. Regression model is plotted as continuous black line, and curved lines indicate confidence intervals (95%) of the mean. Strong evidence was found that multiple climate variables correlate with, and explain, observed variation in ln (HV [μL]) (Table A12).

Increases in exposed temperature records were moderately associated with exponential decreases in mean ln (HV [μL]) (linear regression: *p* < 0.001, F_1,27_ = 40.061, *r*^2^ = 0.597; Figure 7A). Here, 58.2% of observed variation in HV [μL] could be explained by variation in exposed temperature. Regression modelling revealed that exponential increases in HV [μL] were moderately correlated with lower RH. This was only the case when the regression model utilised exposed RH records (linear regression: *p* < 0.001, F_1,27_ = 15.360, *r*^2^ = 0.363; Figure 7B), which indicated that approximately 33.9% of variation observed in log transformed HV [μL] could be explained by exposed RH. Evidence was found to show HV [μL] was moderately correlated against exposed VPD (linear regression: *p* < 0.001, F_1,27_ = 31.823, *r*^2^ = 0.541; Figure 7C), with an estimated 52.4% of variation observed in ln (HV [μL]) explained by exposed VPD. Very strong statistical significance was found in support of the observation that ln (HV [μL]) was negatively associated with mean solar exposure (linear regression: *p* < 0.001, F_1,27_ = 18.976, *r*^2^ = 0.380; Figure 7D). This model had a moderately strong correlation, indicating that approximately 36% of observed variation in ln (HV [μL]) may be explained by variation in fortnightly mean solar exposure.

## 4. Discussion

Broad similarities were identified between Mediterranean and Australian *M. hellenica* populations in the timing of life stages in local seasons [31,53]. As expected, major developmental milestones typically exhibited a six-month delay between Mediterranean and Australian populations. In Australia, the occurrence of life stages between years of study was broadly similar, although the developmental stages showed progressive changes each year; for example, instar stages were detected earlier with successive generations. By contrast, in Greece [50,58], the timing of the occurrence of different instars varied between generations; however, initial detection of each life stage did not occur earlier or later with successive generations. Phenology and development can be delayed or accelerated by changes in climate, a trend observed in other scale insects [10], other Hemiptera [12], and other insect orders [7,53]. Given the relatively short time it has been present, Australian *M. hellenica* may still be adjusting to the effects of climate drivers on their activity. The current study was limited by the fragmented distribution of exotic *P. radiata* in Australia. Relating the seasonal phenology of *M. hellenica* in other climates to that in Australia would provide valuable attestation of the relationships identified here.

The densities of *M. hellenica* on Australian *P. radiata* were typically higher than Mediterranean conspecifics feeding on *Pinus halepensis* during seasonally equivalent months [51]; however, the proportion of honeydew producing insects did not reach proportions as high as those previously documented in Greece [50]. We found evidence that variation in the density of honeydew producing insects and the quantity of honeydew produced could be explained by changes in temperature, humidity, vapour pressure in the atmosphere, and solar exposure. Depending on where climate was recorded (i.e., weather station, exposed tree trunk or bark fissure), the degree of association *M. hellenica* demonstrated with climate variables varied greatly in this study. The insect’s flocculent and preference for crevices and non-sun facing aspects [59] realises a microclimate distinct from broader forest conditions. Microhabitat climate was found to provide weaker predictions of insect activity, as it directly records the climate experienced by sheltered ectothermic *M. hellenica* [37], rather than the experience of the host. With regards to this study, the climate, as experienced by the host, most often provided the best predictive models.

In Australia, variation in the density of *M. hellenica* nymphs and proportion of honeydew producing insects (HPI [%]) were associated with warmer and drier conditions. HPI [%] in Australia was often higher over winter than reported in Greece [50]; however, unlike honeydew availability in Greece, there were no instances where 100% of *M. hellenica* sampled in Australia were excreting honeydew. Broadly, Australian HPI [%] followed similar seasonal patterns to Mediterranean observations, including an increase in honeydew availability following overwintering. Since the phenological study from Greece occurred from 2001 to 2003, it is unclear whether the difference in findings between the current study and previous observations are attributable to underlying geographic differences, inherent generational variation, or host species. The insect density recorded in the current study, from 2019–2020, was only separated by six months from insect densities recorded from Greek conspecifics in 2018–2019 [51]. However, the density of Australian *M. hellenica* was generally higher than the density of the concurrent Greek conspecifics. Insect density, HPI [%], and HPI [cm^−2^] showed a positive relationship with temperature and atmospheric vapour pressure deficit (VPD) and negative association with rainfall and RH. The volume of honeydew produced per insect had the opposite associations with these same climate variables. These relationships with environmental conditions may explain why *M. hellenica* in Australia achieved greater densities than Mediterranean populations. *Marchalina hellenica* in Australia may occupy a more solar exposed, warmer, and drier climate than Mediterranean conspecifics, which are conducive to *Pinus* growth [60,61] and, by association, tree quality as a host for herbivores [62,63]. Unlike previous studies showing microclimate better reflects insect ontogeny, compared to macroclimate records [37], climate conditions within the microhabitat of settled *M. hellenica* nymphs in this study were usually not the best indicator of changes in insect activity. This may be due to strong climate effects on the host’s activity [64,65], and therefore, the availability of resources for *M. hellenica*. The influence of host quality on phloem-feeder activity may therefore be amplified because settled *M. hellenica* are sheltered from direct effects of forest climate condition and depend on climate-driven phloem enrichment [66]. Indeed, phloem-feeder honeydew production and density can fluctuate in association with daily cycles of phloem enrichment [13] and chemical defensive responses [51].

The highest recorded density for Australian *M. hellenica* in this study was over 5 *M. hellenica* per cm^2^, compared to the highest density recorded in Greece of approximately 1.4 *M. hellenica* per cm^2^ [51]. Both of these maximum density records coincided with first instar emergence, and at other times, Greek *M. hellenica* densities were close to zero for most months, and almost always less than 0.25 *M. hellenica* per cm^2^. By contrast, Australian density presented here was 0.5 per cm^2^ or higher across most months. One explanation for elevated *M. hellenica* density in Australia compared to Greece may be that the insect has escaped its natural enemies. The notable enemy is the predatory fly, *Neoleucopis kartliana*, a monophagous predator and prospective biocontrol agent [53,67]. *Neoleucopis kartliana* is recorded as being the most effective and widely distributed predator of *M. hellenica* in Turkey [32]. With *N. kartliana* absent, *M. hellenica* populations in Australia may proliferate with relatively little impact from natural enemies [68,69,70]. If *N. kartliana* is deemed suitable for introduction into Australia as a classical biocontrol agent, inoculative mass-releases of this fly may be guided by the seasonal patterns of Australian *M. hellenica* documented here. For example, the effectiveness of biological control agents can be heavily dependent on the presence of particular life stages, not just any individual, from a given target species [71]. This may be critical to any future release of *N. kartliana*, as the timing of its phenology matches the seasonal phenology of *M. hellenica* [53]. 

Host species is also likely important for *M. hellenica* growth and survival. In the Mediterranean, *Pinus halepensis* is the primary host for *M. hellenica*, a tree that shares a long history of hosting *M. hellenica*, which provide honeydew as an alternative to floral nectar for apicultural purposes [17]. This relationship dates back to the late Roman and early Byzantine Empires along the east coast of the Mediterranean Sea [19,29,72]. A long co-evolutionary history may also be implied by *P. halepensis* possessing targeted defence responses that coincide with seasonal nymph emergence [51,73]. With *M. hellenica* recorded as acquiring the novel host in 2014 [16], *P. radiata* in Australia may be lacking specific defence adaptations that have evolved in *P. halepensis*. Even if *P. radiata* has a similar seasonal-specific defence, the shifting phenology of *M. hellenica* in Australia may allow the insect to escape a temporally restricted defence by emerging earlier. A similar process is documented in other insects (e.g., aphids, bark beetles, Heteroptera), notably involving interactive effects with regional climate [74,75,76] or broadscale climate change [5,42,77].

This study’s findings are similar to previous investigations linking climate to changes in both plant and herbivore activity [76,78,79,80,81]. Climate effects may therefore affect the quality of *M. hellenica* as a host for predatory insects, as has been observed in parasitic/predatory Diptera [82,83]. *Marchalina hellenica* occurs in areas of similar climate conditions to its endemic Greek distribution [67] and is largely dependent on *P. radiata* phloem feeding—the quality of which is influenced by climate conditions [74,84]. For example, VPD is known to be a driver of daily cycles of transpiration and photosynthesis in *Pinus* [40,85,86] and responses to novel climate conditions may alter phloem quality for herbivores. In *Pinus,* these responses are a result of tight regulation of stomatal pore closure and, therefore, strict responses in transpiration, photosynthetic activity, or enrichment of phloem elements [87,88,89]. With such rapid responses to environmental changes, *M. hellenica*, by expropriating carbohydrates, amino acids, and other macromolecules [24], may illicit these strict responses [90] and potentially exacerbate stressful climate effects on the host tree. As global climate conditions deviate from previous means, this insect may experience changes in phenological timing and feeding activity [5,7,91]. Investigation of how *M. hellenica* life cycle and feeding activity may respond to changes to environmental conditions in its endemic geographic range or on native hosts would provide valuable comparisons with the relationships identified in Melbourne populations.

## Data Availability

The data presented in this study are available on request from the corresponding author.

## Appendix A

**Table A1:** Summary statistics of first detections of notable life history milestones and feeding activity across *Marchalina hellenica* instars of a given generation. ± represents one standard deviation.

Generation	Nymph Emergence	1st Ecdysis	2nd Ecdysis	Final Ecdysis	Mean HPI (%)	Max HPI (%)
2018–2019	22 November 2018	11 February 2019	26 April 2019	9 September 2019	30.82 ± 24.66	78.33
2019–2020	18 November 2019	5 February 2020	12 April 2020	28 August 2020	32.82 ± 25.90	95.84
2020–2021	12 November 2019	29 January 2021	8 April 2021	10 September 2021	37.13 ± 22.18	86.80

**Table A2:** Maximum potential temporal range of each *Marchalina hellenica* instar stage and the mean percent of settled insects (SI) and honeydew producing insects (HPI) across phenological records.

Instar Stage	Max Recorded Temporal Range	SI (%)	HPI (%)
1st	12 November–25 February	90.65	33.58
2nd	29 January–7 June	86.88	54.40
3rd	12 April–23 October	73.24	21.81

**Table A3:** Pairwise comparison (equal variances not assumed) to identify if honeydew volume produced by individual *Marchalina hellenica* differed between instar stages. All pairs differences are statistically significant (*p* < 0.05).

Instar Stage	Instar Stage	Mean Diff. (μL)	Std. Error
1	2	0.585	0.119
	3	2.234	0.124
2	1	0.585	0.119
	3	1.658	0.127
3	1	2.243	0.124
	2	1.658	0.127

**Table A4:** Summary of *Marchalina hellenica* honeydew volume produced by instar stage. Sample sizes vary due to differences in honeydew production rates and temporal distribution.

Instar Stage	Max Honeydew (μL)	Mean Honeydew (μL)	Std. Deviation (μL)	N
1st	0.0977	0.019	0.018	98
2nd	0.9688	0.051	0.094	166
3rd	2.2500	0.240	0.342	190

**Table A5:** Summary of Tukey’s HSD post-hoc testing differences in the density of *Marchalina hellenica* between site and generation groups.

Group 1	Group 2	*p*	Mean Difference (cm^−2^)	Standard Error of the Mean
Cardinia 2018	Cardinia 2019	0.998	−0.158	0.349
	Cardinia 2020	0.995	−0.191	0.367
	Dalton Reserve 2018	0.999	−0.211	0.566
	Dalton Reserve 2019	0.999	−0.137	0.356
	Dalton Reserve 2020	0.092	−0.977	0.367
Cardinia 2019	Cardinia 2020	0.999	−0.033	0.272
	Dalton Reserve 2018	0.999	−0.053	0.509
	Dalton Reserve 2019	0.999	0.021	0.256
	Dalton Reserve 2020	0.037	−0.819	0.272
Cardinia 2020	Dalton Reserve 2018	0.999	−0.019	0.522
	Dalton Reserve 2019	0.999	0.054	0.281
	Dalton Reserve 2020	0.092	−0.786	0.295
Dalton Reserve 2018	Dalton Reserve 2019	0.999	0.073	0.514
	Dalton Reserve 2020	0.684	−0.767	0.523
Dalton Reserve 2019	Dalton Reserve 2020	0.039	−0.84	0.281

**Table A6:** Summary of Kruskal-Wallis pairwise comparison testing for differences in the density of honeydew producing nymphs (cm^−2^) between site and generation groups.

Group 1	Group 2	*p*	Mean Difference (cm^−2^)	Standard Error of the Mean	Standardized *t* Statistic
Cardinia 2018	Cardinia 2019	0.999	0.046	10.753	0.914
	Cardinia 2020	0.999	−0.024	10.922	0.242
	Dalton Reserve 2018	0.999	0.012	16.219	0.098
	Dalton Reserve 2019	0.573	−0.167	10.753	2.072
	Dalton Reserve 2020	0.238	−0.192	10.922	2.413
Cardinia 2019	Cardinia 2020	0.999	−0.071	8.546	1.46
	Dalton Reserve 2018	0.999	−0.034	14.724	0.56
	Dalton Reserve 2019	0.002	−0.213	8.329	3.856
	Dalton Reserve 2020	<0.001	−0.238	8.546	4.234
Cardinia 2020	Dalton Reserve 2018	0.999	0.036	14.848	0.284
	Dalton Reserve 2019	0.323	−0.142	8.546	2.299
	Dalton Reserve 2020	0.102	−0.167	8.757	2.708
Dalton Reserve 2018	Dalton Reserve 2019	0.999	−0.179	14.724	1.621
	Dalton Reserve 2020	0.899	−0.204	14.848	1.881
Dalton Reserve 2019	Dalton Reserve 2020	0.999	−0.025	8.546	0.476

**Table A7:** Summary of Kruskal-Wallis pairwise comparison testing for differences in the mean volume of honeydew produced per nymph (μL), between site and generation groups.

Group 1	Group 2	*p*	Mean Difference (μL)	Standard Error of the Mean	Standardized *t* Statistic
Cardinia 2019	Cardinia 2020	0.024	0.112	6.366	2.876
	Dalton Reserve 2019	0.999	−0.28	7.095	0.941
	Dalton Reserve 2020	0.036	0.125	6.366	2.747
Cardinia 2020	Dalton Reserve 2019	<0.001	−0.392	6.154	4.06
	Dalton Reserve 2020	0.999	0.013	5.297	0.155
Dalton Reserve 2019	Dalton Reserve 2020	<0.001	0.405	6.154	3.927

**Table A8:** Summary of Tukey’s Honestly Significant Differences post-hoc analysis of seasonal density collected across 32-months. Only month pairs that demonstrated significant differences in *Marchalina hellenica* density (GPS cm^−2^) are shown (*p* < 0.05). Differences were only detected when comparing November 2020, thus all comparisons shown are comparisons with insect density recorded in November 2020.

Month Compared	Significance Level (*p*)	Mean Difference (GPS [cm^−2^])	Std. Error of Mean
October 2019	0.029	2.915	0.697
January 2020	0.012	3.130	0.697
March 2020	0.041	3.027	0.745
June 2020	0.031	3.106	0.745
September 2020	0.011	3.143	0.697
October 2020	0.006	3.271	0.697

**Table A9:** Summary of climate regression models predicting *Marchalina hellenica* density. Climate records used in models represent the fortnightly mean value of daily maximum, except rainfall; recorded cumulatively in millimetres. N = 34 for each regression analyses.

	Predictor	Model Equation Y =	F	*p*	*r* ^2^	Adj. *r*^2^
Ambient	Temperature [°C]	0.060*x* − 0.261	12.411	<0.001	0.273	0.251
	Solar Exposure [MJ m^−2^]	1.418*x* + 13.982	1.366	0.251	0.040	0.011
Exposed	Temperature [°C]	0.057*x* − 0.340	12.641	<0.001	0.277	0.255
	RH [%]	−0.051*x* + 5.142	18.318	<0.001	0.357	0.337
	VPD [kPa]	0.698*x* + 0.324	10.774	0.002	0.246	0.223
Fissure	Temperature [°C]	0.870*x* − 0.687	17.164	<0.001	0.342	0.322
	RH [%]	−0.041*x* + 4.396	4.883	0.034	0.129	0.103
	VPD [kPa]	1.591*x* + 0.235	9.456	0.004	0.223	0.199
Melb. Water	Rainfall [mm]	−0.010*x* + 0.871	3.101	0.088	0.086	0.058

**Table A10:** Summary of climate regression models predicting the percentage of honeydew producing *Marchalina hellenica*. Climate records used in models represent the fortnightly mean value of daily maximum, except rainfall; recorded cumulatively in millimetres. N = 30 for each regression analyses.

	Predictor	Model Equation Y =	F	*p*	*r* ^2^	Adj. *r*^2^
Ambient	Temperature [°C]	0.028*x* − 0.118	19.177	<0.001	0.398	0.377
	Solar Exposure [MJ m^−2^]	0.018*x* + 0.079	8.414	0.007	0.225	0.198
Exposed	Temperature [°C]	0.022*x* − 0.077	10.617	0.003	0.298	0.243
	RH [%]	−0.012*x* + 1.355	4.706	0.038	0.140	0.110
	VPD [kPa]	0.215*x* + 0.208	4.293	0.047	0.129	0.099
Fissure	Temperature [°C]	0.031*x* − 0.166	11.296	0.002	0.280	0.256
	RH [%]	−0.011*x* + 1.305	1.951	0.173	0.063	0.031
	VPD [kPa]	0.42*x* + 0.195	2.976	0.095	0.093	0.062
Melb. Water	Rainfall [mm]	0.001*x* + 0.289	0.048	0.828	0.002	0.033

**Table A11:** Summary of climate regression models predicting log (mean HPI density [no cm^−2^]). Climate records used in models represent the fortnightly mean value of daily maximum, except rainfall; recorded cumulatively in millimetres. N = 30 for each regression analysis. The absence of autocorrelation of the data is rejected if the Approximate Nonlinear Durbin-Watson (AND) statistic is less than 0.622 or greater than 2.041.

	Predictor	Model Equation Y =	F	*p*	*r* ^2^	*Adj. r* ^2^	AND
Ambient	Temperature [°C]	0.180x − 4.711	35.231	<0.001	0.549	0.533	1.136
	Solar Exposure [MJ m^−2^]	0.106x − 3.315	10.122	0.003	0.259	0.233	0.633
Exposed	Temperature [°C]	0.161x − 4.746	25.174	<0.001	0.465	0.446	0.993
	RH [%]	−0.097x + 6.536	12.858	0.0013	0.303	0.279	0.754
	VPD [kPa]	1.98x − 2.858	16.317	<0.001	0.360	0.338	0.981
Fissure	Temperature [°C]	0.23x − 5.483	30.523	<0.001	0.513	0.496	1.194
	RH [%]	−0.082x + 5.466	3.772	0.062	0.115	0.085	0.509
	VPD [kPa]	3.847x − 2.975	10.081	0.004	0.258	0.232	0.734
Melb. Water	Rainfall [mm]	−0.014x − 1.727	0.939	0.340	0.031	0.002	0.534

**Table A12:** Summary of climate regression models predicting log (mean volume of honeydew produced per *M. hellenica* sampled), with zeros removed. Climate records used in models represent the fortnightly mean value of daily maximum, except rainfall; recorded cumulatively in millimetres. N = 28. The absence of autocorrelation of the data is rejected if the Approximate Nonlinear Durbin-Watson (AND) statistic is less than 0.566 or greater than 2.098.

	Predictor	Model Equation Y =	F	*p*	*r* ^2^	Adj. *r*^2^	AND
Ambient	Temperature [°C]	−0.183*x* + 0.511	34.286	<0.001	−0.559	0.543	1.041
	Solar Exposure [MJ m^−2^]	−0.122*x* − 0.729	15.591	<0.001	0.366	0.343	0.750
Exposed	Temperature [°C]	−0.187*x* + 0.928	40.061	<0.001	0.597	0.582	0.878
	RH [%]	0.106*x* − 11.643	15.360	<0.001	0.363	0.339	0.781
	VPD [kPa]	−2.543*x* − 1.157	31.823	<0.001	0.541	0.524	0.944
Fissure	Temperature [°C]	−0.242*x* + 1.427	30.820	<0.001	0.533	0.516	0.903
	RH [%]	0.041*x* − 5.970	0.858	0.362	0.031	0.005	0.498
	VPD [kPa]	−3.768*x* − 1.278	8.337	0.008	0.236	0.208	0.580
Melb. Water	Rainfall [mm]	−0.007*x* − 2.073	0.210	0.650	0.008	0.029	0.507
